# Defining levels of care in cardiogenic shock

**DOI:** 10.3389/fcvm.2023.1206570

**Published:** 2023-10-31

**Authors:** Miguel Alvarez Villela, Danni Fu, Kylie Roslin, Rebecca Smoller, Daniel Asemota, Daniel J. Miklin, Arber Kodra, Sirish Vullaganti, Robert O. Roswell, Sabarivinoth Rangasamy, Christina E. Saikus, Zachary N. Kon, Matthew J. Pierce, Gregg Husk, Gerin R. Stevens, Simon Maybaum

**Affiliations:** ^1^ Department of Cardiology, Lenox Hill Hospital, Northwell Health, New York, NY, United States; ^2^ Department of Cardiology, Northshore University Hospital, Northwell Health, Manhasset, NY, United States; ^3^ Department of Medicine, Lenox Hill Hospital, Northwell Health, New York, NY, United States; ^4^ Department of Cardiology, Northern Westchester Hospital, Northwell Health, Mount Kisco, NY, United States; ^5^ Department of Cardiothoracic Surgery, Northshore University Hospital, Northwell Health, Manhasset, NY, United States

**Keywords:** cardiogenic shock, acute myocardial infarction, acute heart failure, mechanical circulatory support, levels of care, cardiogenic shock centers

## Abstract

**Background:**

Expert opinion and professional society statements have called for multi-tier care systems for the management of cardiogenic shock (CS). However, little is known about how to pragmatically define centers with different levels of care (LOC) for CS.

**Methods:**

Eleven of 23 hospitals within our healthcare system sharing a common electronic health record were classified as different LOC according to their highest mechanical circulatory support (MCS) capabilities: Level 1 (L-1)—durable left ventricular assist device, Level 1A (L-1A)—extracorporeal membrane oxygenation, Level 2 (L-2)—intra-aortic balloon pump and percutaneous ventricular assist device; and Level 3 (L-3)—no MCS. All adult patients treated for CS (International Classification of Diseases, ICD-10 code R57.0) between 2016 and 2022 were included. Etiologies of CS were identified using associated diagnostic codes. Management strategies and outcomes across LOC were compared.

**Results:**

Higher LOC centers had higher volumes: L-1 (*n* = 1): 2,831 patients, L-1A (*n* = 4): 3,452, L-2 (*n* = 1): 340, and L-3 (*n* = 5): 780. Emergency room admissions were more common in lower LOC (96% at L-3 vs. 46% L-1; *p* < 0.001), while hospital transfers were predominant at higher LOC (40% at L-1 vs. 2.7% at L-3; *p* < 0.001). Men comprised 61% of the cohort. Patients were younger in the higher LOC [69 (60–78) years at L-1 vs. 77 (67–85) years at L-3; *p* < 0.001]. Patients with acute myocardial infarction (AMI)-CS and acute heart failure (AHF)-CS were concentrated in higher LOC centers while other etiologies of CS were more common in L-2 and L-3 (*p* < 0.001). Cardiac arrest on admission was more prevalent in lower LOC centers (L-1: 2.8% vs. L-3: 12.1%; *p* < 0.001). Patients with AMI-CS received more percutaneous coronary intervention in lower LOC (51% L-2 vs. 29% L-1; *p* < 0.01) but more coronary arterial bypass graft surgery at higher LOC (L-1: 42% vs. L-1A: 23%; *p* < 0.001). MCS use was consistent across levels for AMI-CS but was more frequent in higher LOC for AHF-CS patients (L-1: 28% vs. L-2: 10%; *p* < 0.001). Despite increasing in-hospital mortality with decreasing LOC, no significant difference was seen after multivariable adjustment.

**Conclusion:**

This is the first report describing a pragmatic classification of LOC for CS which, based on MCS capabilities, can discriminate between centers with distinct demographics, practice patterns, and outcomes. This classification may serve as the basis for future research and the creation of CS systems of care.

## 1. Introduction

Cardiogenic shock (CS) is caused by severe impairment in myocardial performance that leads to diminished cardiac output, end-organ hypoperfusion, multi-organ failure, and death. Acute coronary syndrome (ACS) is the most well recognized etiology of CS, but decompensated heart failure (HF) and other non-ischemic etiologies are being increasingly recognized as common, comprising up to 30%–50% of cases in certain hospital settings in North America (1). Despite advancements in treatment including growing access to primary percutaneous coronary interventions (PCI) and mechanical circulatory support (MCS) devices, 30-day mortality remains high (2). However, outcomes vary across different hospital settings in the United States, with lower mortality reported in larger urban and left ventricular assist device (LVAD)–capable centers compared with smaller, rural hospitals (3).

This difference in outcomes could be related to variations in management patterns across different types of centers (4). For instance, the use of MCS seems to be higher among patients treated in larger hospitals compared to those admitted to smaller ones (5). Accordingly, over 80% of venoarterial extracorporeal membrane oxygenation (VA-ECMO) cases are performed in large, urban, teaching hospitals (6).

In order to standardize care for CS patients and improve outcomes, there have been multiple calls from professional societies and expert opinion papers for the creation of multi-level systems of care that allow for the rapid triage and transfer of complex CS patients toward centers with higher volume, greater familiarity with advanced MCS, and built-in multidisciplinary teams (4, 7, 8).

However, there is no standardized classification system for hospital tiers in the management of CS and a pragmatic definition of levels of care (LOC) is yet to be established (8).

One important barrier to creating this definition is the lack of systematic accounts of CS epidemiology and management within existing integrated multi-level healthcare systems, which could serve as the basis for a new model. Most of the current CS registries are comprised almost exclusively of patients treated primarily at high-tier centers and patients who were transferred to these centers from local spoke hospitals for definitive treatment (9, 10).

Northwell Health is a multi-tier hospital system in the greater New York metropolitan area. Although care is integrated within the system and transfers toward higher tier centers are common, a cardiogenic shock team to formally triage these patients was not established at our quaternary, durable LVAD and orthotropic heart transplant (OHT) center, until 2020. Shock teams were otherwise not available at any other centers until late 2022.

Herein we present the epidemiology, management, and outcomes of patients with CS treated across all tiers within our system in the last 6 years and propose a pragmatic definition of CS LOC.

## 2. Methods

This is a multi-center retrospective observational study approved by Northwell Health Institutional Review Board with ID number 22-0834. There was no greater than minimal risk to the study subjects and waiver of informed consent was obtained. Patients were de-identified and data were stored in a secure password protected database.

### 2.1. Study centers and level of care definitions

Our cohort comprised 11 hospitals sharing a common electronic health record (Sunrise Clinical Manager) including all cardiac surgery centers within our 23-hospital consortium in the New York metropolitan area. Each level is organized by virtue of the highest MCS capability available.

Level 1 (L-1) is the durable LVAD and heart transplant capable center. Level 1A (L-1A) hospitals have the capability to offer all temporary MCS, including venoarterial ECMO. Level 2 (L-2) hospitals can offer intra-aortic balloon pump (IABP) and percutaneous ventricular assist device (pVAD) but not ECMO and Level 3 (L-3) hospitals do not have an onsite cardiac catheterization laboratory (CCL) and do not offer temporary MCS or PCI. Both, L-1 and L-1A had onsite cardiac surgery for the duration of the study. A Level 2 center had primary PCI capabilities but no onsite cardiac surgery. Bed capacity at L-1 was 756, among L-1A centers ranged from 313 to 807, at L-2 was 348, and L-3 ranged from 103 to 312. A full center capability description is outlined in Supplementary Table S3.

All hospitals were located within the greater New York Metropolitan area. The longest linear distance in our cohort between an L-1A center and an L-1 center was 24 miles, between an L-2 and an L-1 center was 20.2 miles, and between an L-3 and an L-1 center was 15.7 miles. In our healthcare system, inclusive of hospitals not included in this data cohort, the longest linear distance between any hospital and an L-1 center was 56 miles.

We describe the epidemiology, practice patterns, and in-hospital outcomes for each of these levels.

### 2.2. Patient population and data collection

We included patients over the age of 18 who were discharged with dead or alive status from all sites in the Northwell Health System using Sunrise Clinical Manager electronic health record between January 2016 and August 2022. CS was identified using the International Classification of Diseases (ICD)-10 code R57.0 as a principal or secondary diagnosis on discharge.

Data including admission type, transfer among hospitals, demographics, major comorbidities, primary insurance carrier, length of stay (LOS), admission diagnosis, principal diagnosis, secondary diagnosis, comorbidities, and all procedures of interest performed during the hospital stay were collected. Comorbidities were summarized using the ICD-10 version of the Charlson Comorbidity Index (CCI) (11). Patients who were transferred into each hospital level were included, independent of whether they came from hospitals within our cohort or outside of it. Each admission to each hospital was considered independently if the transfer happened within our hospital cohort. Data on procedures performed for patients transferred from one center to another were counted as part of the procedural volume for the center where they were performed. For example, in the case of a patient with AMI-CS who underwent diagnostic left heart catheterization (LHC) and IABP placement at an L-2 center and was transferred to the L-1 center for coronary arterial bypass graft (CABG) surgery, the LHC and IABP will be counted as part of the L-2 volume and the CABG as part of the L-1 center volume. Data on procedures performed at transferring centers outside the cohort were not available, and in this case, only procedures performed at the receiving center were included. Patients who were readmitted were analyzed as individual patients for each admission. A transfer map was created to represent the patterns of flow for CS patients within our system.

The complete list of procedure codes considered can also be found in the Supplementary Material (Supplementary Table S2).

### 2.3. Shock etiologies

The etiology of CS was classified into three major categories based on associated principal or secondary discharge diagnosis: (1) acute myocardial infarction (AMI) including ST-elevation myocardial infarction (STEMI) and non-ST-elevation myocardial infarction (NSTEMI); (2) acute heart failure (AHF) including decompensated heart failure, arrhythmia, and valvular disease; (3) Other including, for example, sepsis and pulmonary embolism (Supplementary Figure 1).

A complete list of ICD-10 codes included in each etiologic category is available in the Supplementary Material.

Discharge status was used to identify in-hospital outcomes and was divided into four major categories: (1) death; (2) transfer to another hospital; (3) discharge home or to a skilled nursing facility, inpatient rehabilitation facility, or requiring home care; (4) transferred to hospice facility.

### 2.4. Statistical analysis

Data analysis was done using the GraphPad PRISM software. Continuous variables were reported as mean and standard deviation or median and interquartile range, and categorical variables were reported as number (percentage). Comparisons of quantitative variables (age and LOS) were analyzed using the non-parametric Kruskal–Wallis tests and *post-hoc* Dunn tests.

Categorical variables were assessed with chi-squared analysis or Fisher's exact tests depending on parametric distribution. *p*-values <0.05 were considered statistically significant. (*p*-values are for trends across levels of care unless otherwise indicated.)

Multivariate linear and logistic regression was used to compare survival to hospital discharge between levels of care, adjusting for potential confounders, which included age, sex, Charlson Comorbidity Index, insurance type, and etiology of CS.

## 3. Results

### 3.1. General data trends

Our cohort comprised one L-1 center, four L-1A centers, one L-2 center, and five L-3 centers. During the study period, a total of 7,402 patients were treated within our hospital cohort: The higher LOC contributed the largest number of patients: L-1 treated 2,830, L-1A 3,452 patients, L-2 340 patients, and L-3 780 patients.

**Figure 1:**
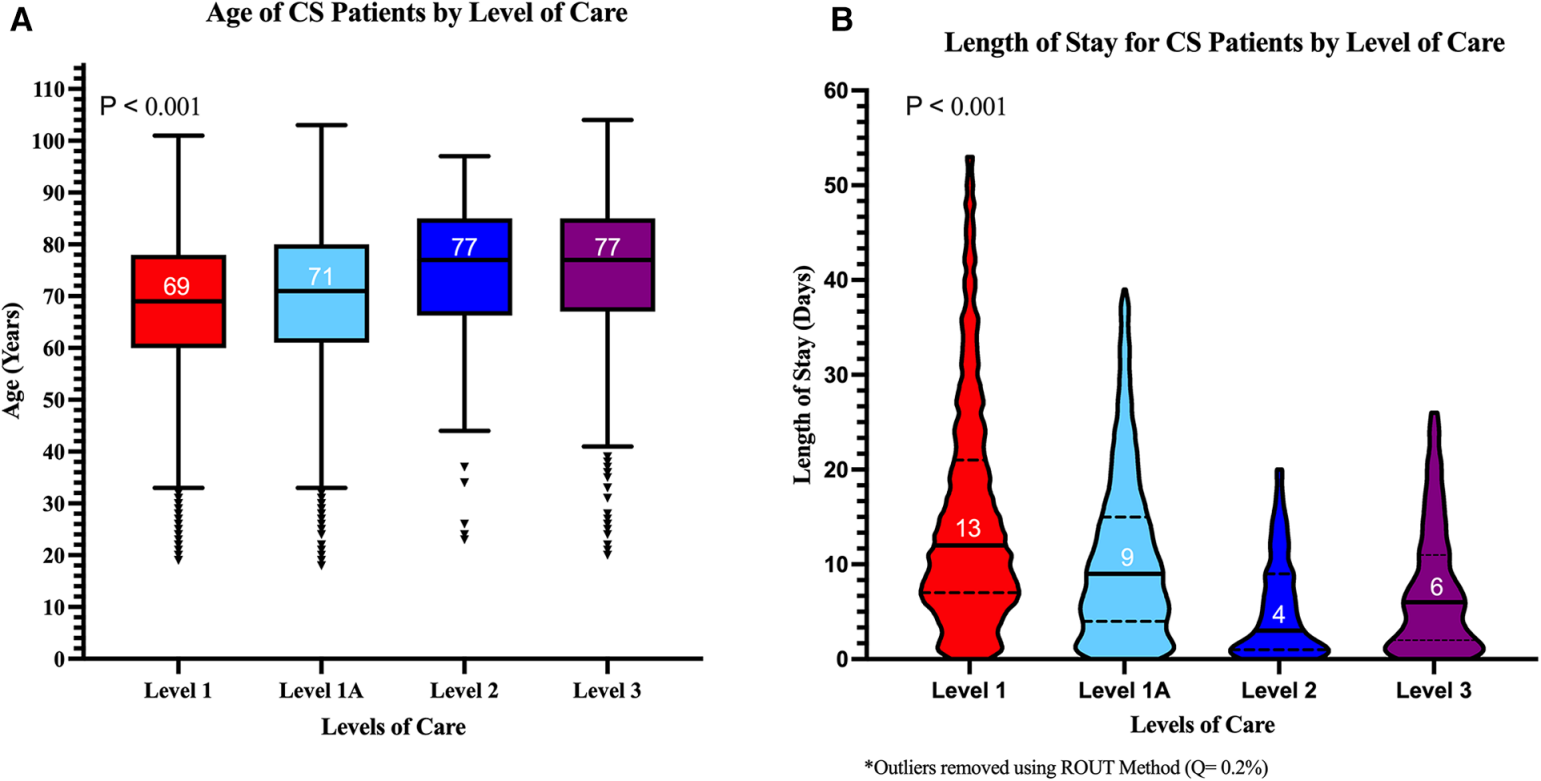
Median age and length of stay for cardiogenic shock patients across levels of care. (**A**) Median age was higher in lower LOC. (**B**) Median length of stay was longer in higher LOC. The *p*-values shown represent trends across LOC.

Men were a majority (61%) overall but were less prevalent in the lower LOC (only 53% at L-3; *p* < 0.001) (Table 1 and Figure 1). Patients were older in the lower LOC centers while women were older than men across all LOC (*p* < 0.001) (Supplementary Figure 2).

**Table 1:** Baseline characteristics across LOC.

	Level 1 (*n* = 2,830)	Level 1A (*n* = 3,452)	Level 2 (*n* = 340)	Level 3 (*n* = 780)	*p*-value
Age (median, IQR)	69 (60–78)	71 (61–80)	77 (67–85)	77 (67–85)	<0.0001
Length of stay, days (median, IQR)	13 (7–24)	9 (4–16)	4 (1–10)	6 (2–13.7)	<0.0001
Sex, male, *n* (%)	1,796 (63.4%)	2,149 (62.3%)	209 (61.5%)	416 (53.3%)	<0.0001
Insurance					<0.0001
Medicare/Medicaid (%)	86.4%	89.1%	92.1%	92.6%	
Private (%)	11.8%	9.8%	6.2%	6.2%	
Other (%)	1.8%	1.2%	1.5%	1.3%	
HTN, *n* (%)	1,967 (69.5%)	2,143 (62.1%)	175 (51.5%)	578 (74.1%)	<0.0001
CKD, *n* (%)	938 (33.1%)	959 (27.8%)	87 (25.6%)	237 (30.4%)	<0.0001
HF, *n* (%)	1,623 (57.3%)	1,801 (52.2%)	134 (39.4%)	438 (56.2%)	<0.0001
CAD, *n* (%)	1,339 (47.3%)	1,254 (36.3%)	115 (33.8%)	220 (28.2%)	<0.0001
DM2, *n* (%)	1,218 (43.0%)	1,305 (37.8%)	85 (25.0%)	323 (41.4%)	<0.0001
Atrial fibrillation, *n* (%)	1,177 (41.6%)	1,189 (34.4%)	106 (31.2%)	310 (39.7%)	<0.0001
VTE, *n* (%)	64 (2.3%)	39 (1.1%)	2 (0.6%)	6 (0.8%)	0.0003
Anemia, *n* (%)	1,018 (36.0%)	1,089 (31.5%)	76 (22.4%)	295 (37.8%)	<0.0001
Cardiac arrest at admission, *n* (%)	78 (2.8%)	165 (4.8%)	45 (13.2%)	94 (12.1%)	<0.0001
Admission type					
Emergency room, *n* (%)	1,304 (46.1%)	2,547 (73.8%)	331 (97.4%)	748 (95.9%)	<.0.0001
Transfer in	1,129 (40%)	583 (17%)	3 (0.9%)	21 (2.7%)	<.0.0001
Other	396 (14.0%)	322 (9.3%)	6 (1.7%)	11 (1.4%)	<0.0001
Shock etiologies					
AMI	887 (31.3%)	961 (27.8%)	108 (31.8%)	66 (8.5%)	<0.0001
AHF	971 (34.3%)	1,089 (31.5%)	59 (17.4%)	137 (17.6%)	<0.0001
Other (%)	972 (34.3%)	1,402 (40.6%)	173 (50.9%)	577 (74.0%)	<0.0001
Sepsis (% of “other”)	417 (42.9%)	628 (44.8%)	81 (46.8%)	320 (55.5%)	<0.0001
Pulmonary embolism (% of “other”)	45 (4.6%)	37 (2.6%)	4 (2.3%)	14 (2.4%)	0.0245
Other non-cardiac (% of “other”)	300 (30.9%)	367 (26.2%)	55 (31.8%)	128 (22.2%)	<0.0009

CAD, coronary artery disease; CKD, chronic kidney disease; DM2, diabetes mellitus type 2; HTN, hypertension; VTE, venous thromboembolism.

**Figure 2:**
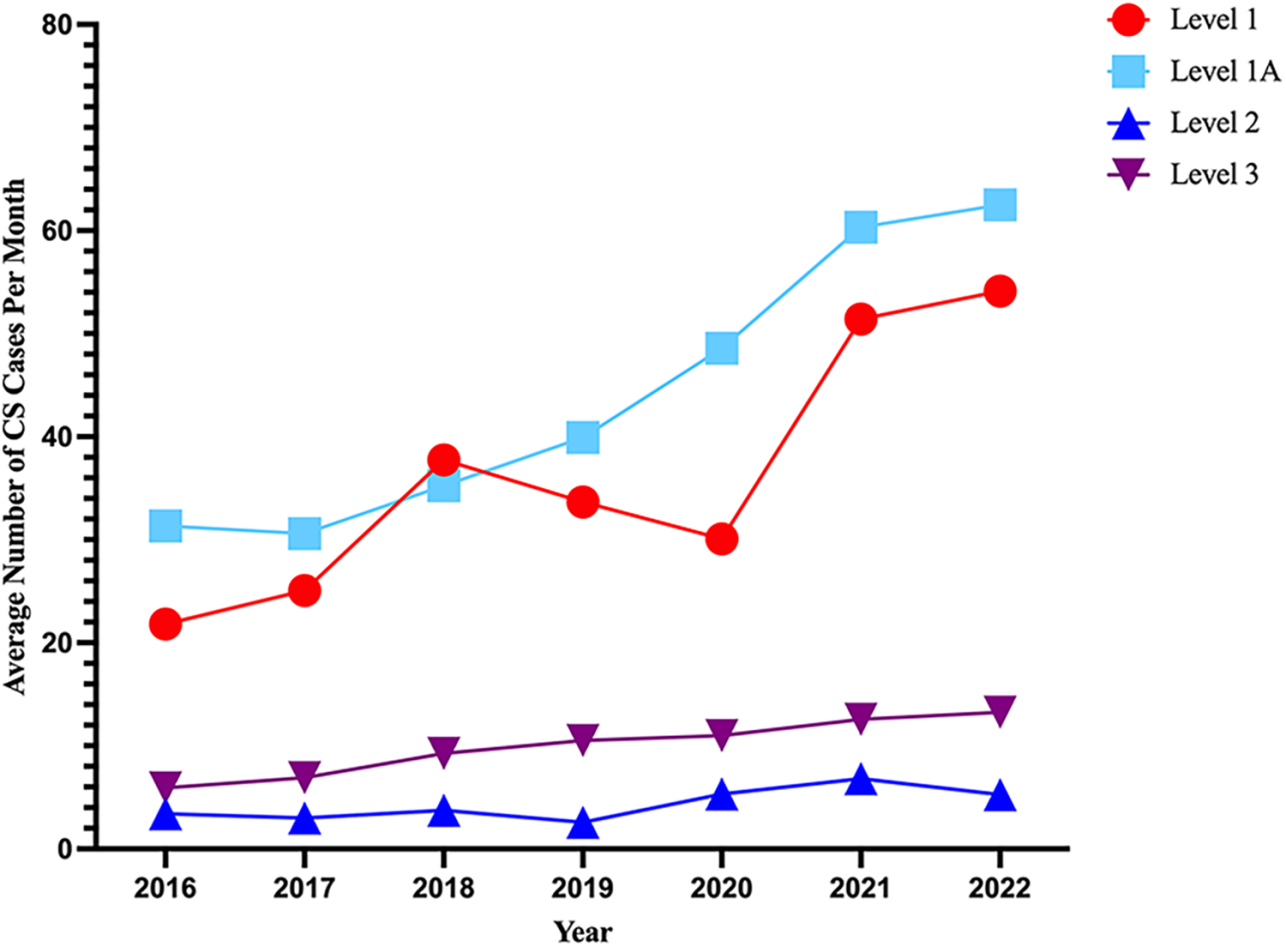
Prevalence of cardiogenic shock by levels of care over time. Average number of patients with either a principal or secondary diagnosis of cardiogenic shock (ICD-10 code R57.0) in each year from 2016 up until August 2022.

**Figure 3:**
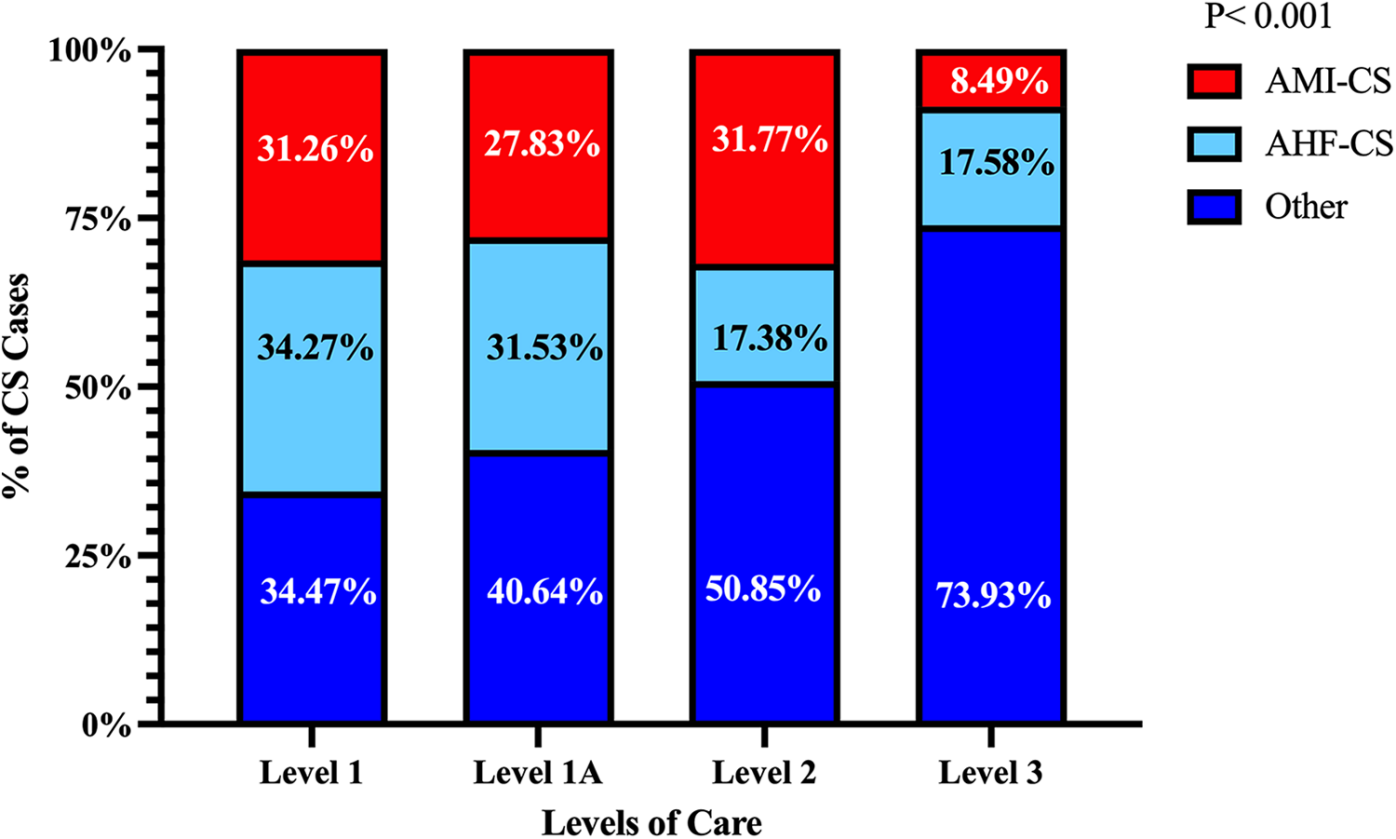
Relative prevalence of each cardiogenic shock etiology across levels of care. AMI-CS and AHF-CS were more prevalent in the higher LOC while “other” etiologies of CS were more prevalent in the lower LOC. The *p*-value shown is for trend across LOC.

The average number of CS cases increased over the study period for L-1 and L-1A but remained mostly unchanged at L-2 and L-3 hospitals (Figure 2). The overall prevalence of each CS etiology was AMI 27%, AHF 30%, and “other” 42.2%. In the “other” category, the most common principal diagnosis was sepsis (49%). The relative prevalence of each CS etiology varied significantly across hospital levels as shown in Figure 3. The prevalence of AMI-CS was similar across centers with cardiac catheterization laboratories and much lower at L-3 while AHF-CS patients were concentrated in the durable LVAD- and ECMO-capable centers (L-1 and L-1A).

Notably, the prevalence of the “other” etiologic category increased substantially in the lower levels of care. Admission types varied across LOC. Patients in L-1 and L-1A were more frequently transferred from other centers, while patients in L-2 and L-3 were more commonly admitted through the emergency room (ER) (Table 1). L-1 was the highest receiver of transfers followed by L-1A. Patients arriving as inter-hospital transfers to these LOC most often came from hospitals outside of our cohort. In L-1, most transfers from within our hospital cohort came from L-1A centers (68%), while 10% came from L-2 and 21% came from L-3. Most patients received as transfers had a diagnosis of AHF-CS (45%), while AMI-CS (28%) and other CS etiologies (14%) were less common.

**Figure 4:**
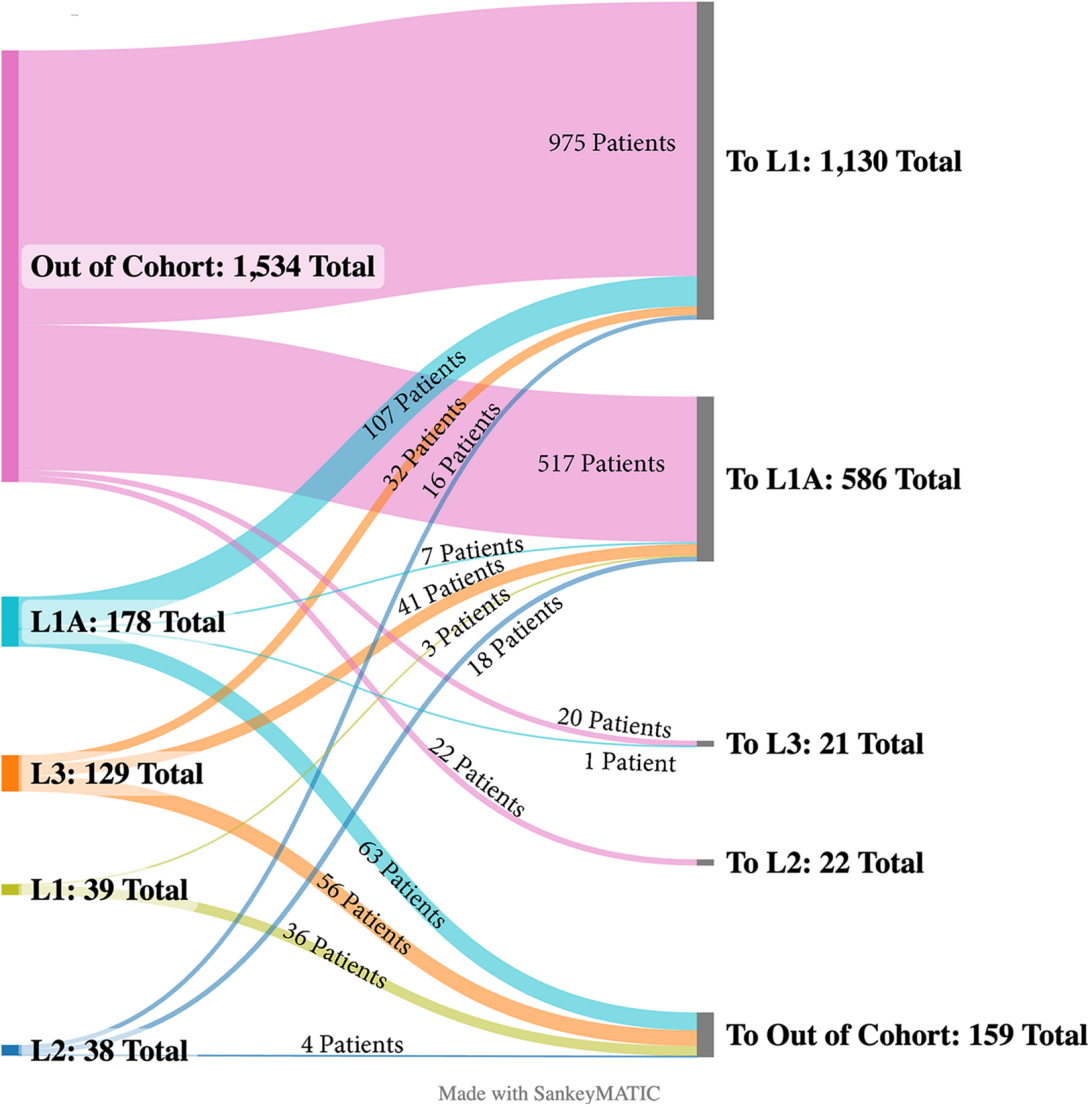
Inter-hospital transfer map. Sankey diagram displaying the transfer of CS patients from their original level of care to their destination level.

In L-2, 17% of patients were transferred out to other hospitals: 24% of AMI-CS patients, 32% of AHF-CS patients, and 6.4% for those with other etiologies of CS. In L-3, patients were also transferred out 17% of the time: AMI-CS patients 29%, AHF-CS 26%, and patients with other etiologies 13%. Figure 4 depicts the flow of transfers across LOC.

Patients transferred to the higher LOC were significantly younger than the average patients at those centers: Level 1, 66 years (57–75) vs. 69 years (60–78) (*p* < 0.0001) and Level 1A, 69 years (58–78) vs. 71 years (61–80) (*p* = 0.0007).

The median LOS was shorter in the lower LOC, possibly reflecting an effect of inter-hospital transfers with patients with the higher complexity moving toward the durable LVAD- and ECMO-capable centers (*p* < 0.001) (Figure 1B).

Cardiac arrest on admission to initial hospital, an important risk modifier in CS, was significantly more common among patients admitted to lower LOC: 12% in L-3 and 13% in L-2 vs. 2.8% at L-1 (Table 1).

The prevalence of relevant comorbidities and the Charlson Comorbidity Index are listed in Table 1. Non-cardiac conditions such as chronic kidney disease (CKD), diabetes mellitus, and anemia were highly prevalent in all LOC.

**Figure 5:**
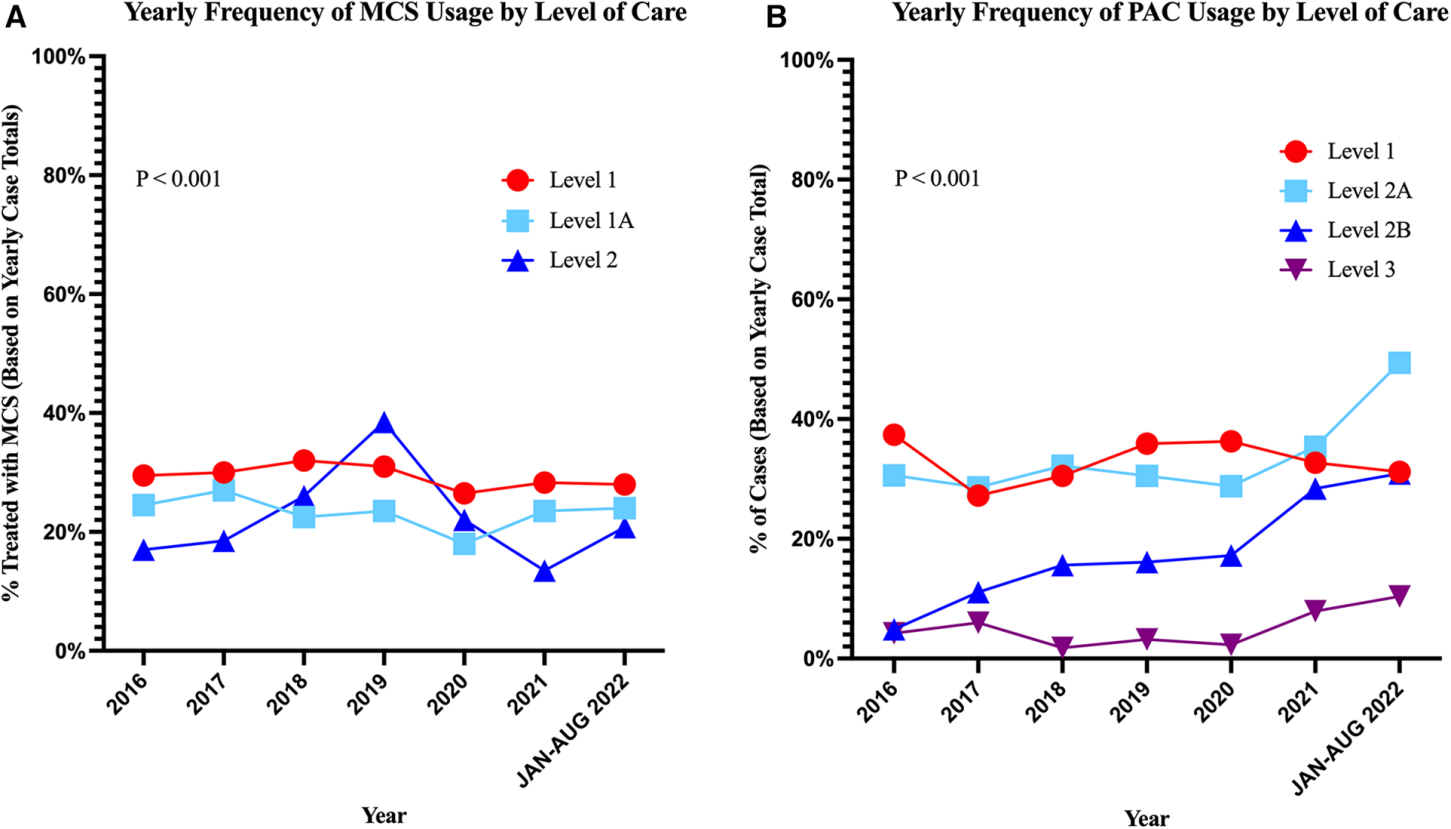
Frequency of mechanical circulatory support and pulmonary artery catheter use over time across levels of care. (**A**) Trends in overall MCS use per LOC. (**B**) Trends in pulmonary artery catheter use over time by LOC. The *p*-values shown represent overall difference in MCS or PAC usage across LOC.

The overall use of MCS decreased with decreasing LOC (Table 2, *p* < 0.001) and these trends remained stable over time (Figure 5A), while the overall use of pulmonary artery catheter (PAC) was high at L-1 and L-1A but much lower in L-2 and was rare in L-3 (*p* < 0.001) (Figure 5B). The frequency of renal replacement therapies and invasive and non-invasive ventilation across LOC is detailed in Table 2.

**Table 2:** Management procedures.

	Level 1 (*n* = 2,830)	Level 1A (*n* = 3,452)	Level 2 (*n* = 340)	Level 3 (*n* = 780)	*p*-value
Overall MCS use	29.5%	22.9%	18.4%	NA	<0.0001
Overall PAC use	32.9%	34.0%	19.1%	5.1%	<0.0001
Renal replacement therapy	18.8%	17.1%	8.8%	11.7%	<0.0001
Mechanical ventilation	45.4%	52.8%	55.6%	57.2%	<0.0001
Non-invasive mechanical ventilation	20.4%	22.8%	9.4%	23.2%	<0.0001
AMI	*n* = 887 (31%)	*n* = 961 (28%)	*n* = 108	*n* = 66	
LHC *n* (%)	501 (56.5%)	659 (68.6%)	79 (73.1%)	NA	<0.0001
PCI	256 (28.9%)	418 (43.5%)	55 (50.9%)	NA	<0.0001
CABG	368 (41.5%)	223 (23.2%)	NA	NA	<0.0001
Valvular procedures	127 (14.3%)	56 (5.8%)	NA	NA	<0.0001
Total MCS	474 (53.4%)	546 (56.8%)	62 (57.4%)	NA	0.3118 (NS)
PAC	344 (39.2%)	450 (46.8%)	23 (21.3%)	1 (1.5%)	<0.0001
AHF	*n* = 971 (34%)	*n* = 1,089 (32%)	*n* = 59	*n* = 137	
LHC	273 (28.1%)	292 (26.8%)	11 (18.6%)	NA	0.2625 (NS)
PCI	23 (2.4%)	48 (4.4%)	1 (1.7%)	NA	0.0296
CABG	73 (7.5%)	49 (4.5%)	NA	NA	0.0049
Valvular procedures	282 (29.0%)	148 (13.6%)	NA	NA	<0.0001
Total MCS	277 (28.5%)	156 (14.3%)	6 (10.2%)	NA	<0.0001
PAC	448 (46.1%)	433 (39.8%)	10 (16.9%)	7 (5.1%)	<0.0001
Other etiologies	*n* = 972 (34%)	*n* = 1,402 (41%)	*n* = 173	*n* = 577	
LHC	90 (9.3%)	162 (11.6%)	18 (10.4%)	NA	0.2019 (NS)
PCI	12 (1.2%)	49 (3.5%)	4 (2.3%)	NA	0.0027
CABG	24 (2.5%)	31 (2.2%)	NA	NA	0.6798 (NS)
Valvular procedures	54 (5.6%)	76 (5.4%)	NA	NA	0.9270 (NS)
Total MCS	85 (8.7%)	88 (6.3%)	4 (2.3%)	NA	0.0031
PAC	139 (14.3%)	290 (20.7%)	32 (18.5%)	32 (5.5%)	<0.0001

### 3.2. Management strategies by etiology and level of care

#### 3.2.1. AMI-CS

**Figure 6:**
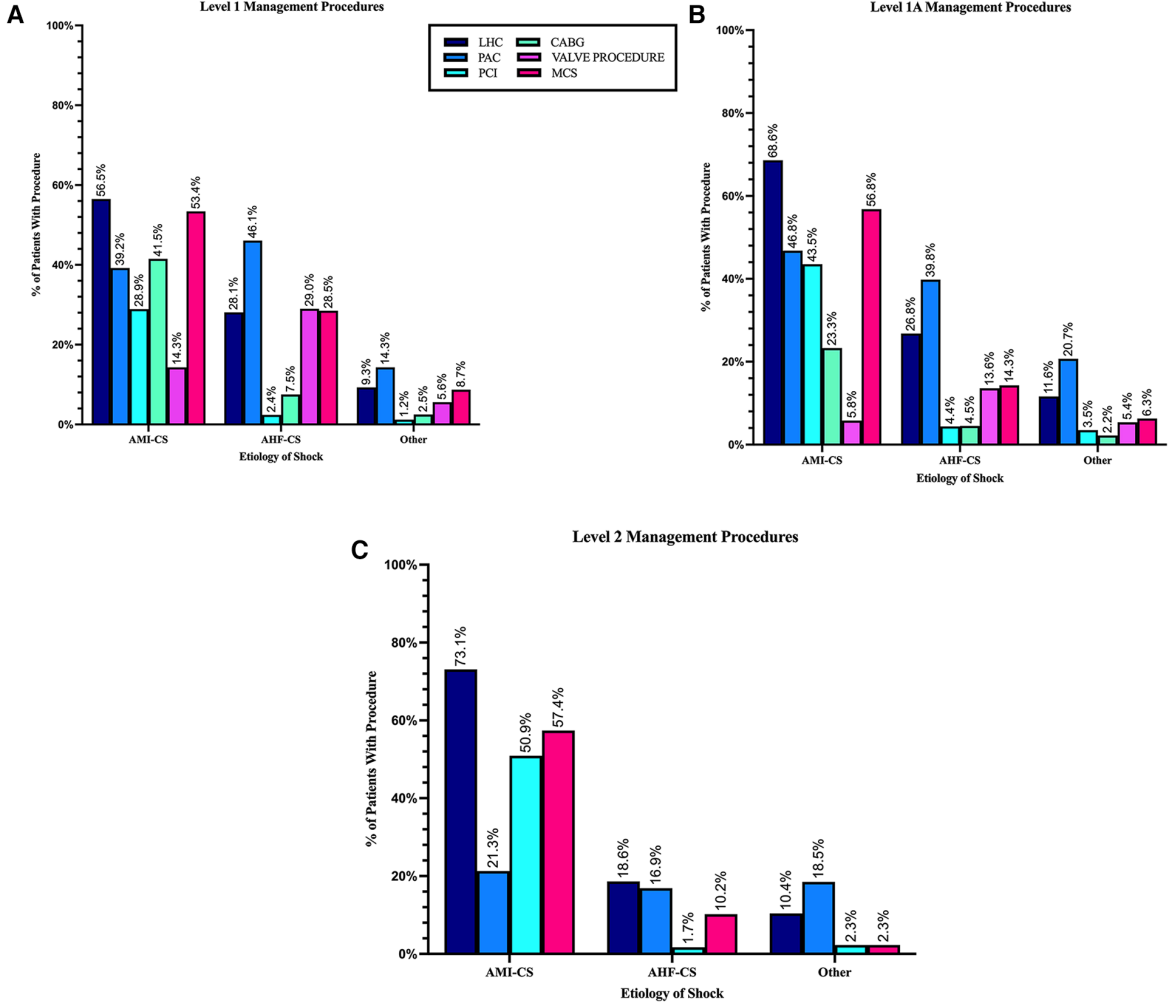
Management procedures by cardiogenic shock etiology in each level of care. (**A**) Level 1 LVAD-capable center. (**B**) Level 1A ECMO-capable centers. (**C**) Level 2 pVAD-capable center. Level 3 not depicted since no interventional or surgical procedures are performed at this level.

The use of LHC and PCI were higher in L-1A (69% and 43%) and L-2 (73% and 51%) than L-1 (57% and 29%) where CABG (41.5%) and surgical and transcatheter valve procedures (14.3%) were more frequent (Table 2 and Figure 6A).

MCS use in patients with AMI-CS was similar across capable LOC (L-1 to L-2) (Table 2, *p* = 0.3). However, the use of PAC to guide management was higher in L1 (39%) and L-1A (47%) than in L2 (21%) (*p* < 0.001) (Table 2 and Figure 6).

#### 3.2.2. AHF-CS

Across LOC, the treatment of AHF-CS was less uniform than for AMI-CS patients. The largest difference was seen in the use of MCS. While it was used less frequently than in patients AMI-CS overall, its use in L-1 (28%) was close to double what it was in L-1A (14%) and close to three times as high as in L-2 (10%) (*p* < 0.001).

While LHC was performed at similar rates in L-1, L-1A, and L-2, the use of PAC declined significantly in lower LOC with almost half of the patients treated with it in L-1 (46%) but only a fraction at L-2 (17%) and L-3 (5%).

Valvular procedures (29% vs. 14%) and CABG (7.5% vs. 4.5%) were significantly more common in L-1 compared to L-1A (*p* < 0.05), both levels with cardiac surgery capabilities (Table 2 and Figure 6).

#### 3.2.3. Other etiologies of CS

This group comprised a variety of etiologies with the most prevalent being sepsis, possibly indicating the presence of mixed cardiogenic and septic shock; other etiologies are detailed in Supplementary Figure 1.

Patients in this group underwent the lowest rates of cardiac procedures across all LOC, including LHC, PCI, CABG, and valvular procedures. PAC placement was the most frequently performed procedure with higher use in higher LOC (*p* < 0.001). Although rare, MCS use was also more frequent in the higher LOC within this patient group (*p* < 0.001).

### 3.3. In-hospital outcomes

**Figure 7:**
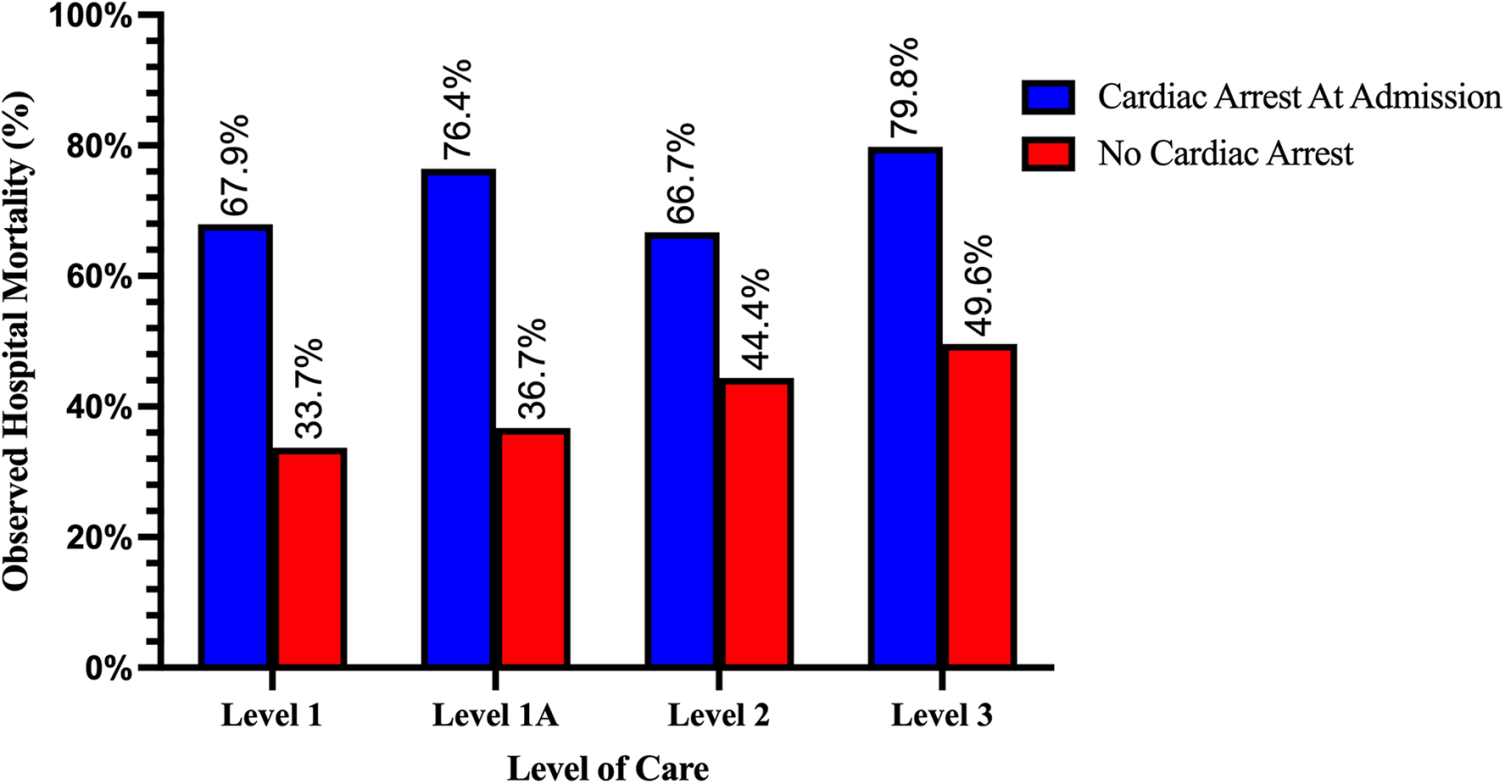
Observed mortality rate among cardiogenic shock patients with and without cardiac arrest on admission by level of care. Mortality increased in a stepwise fashion with decreasing LOC and was higher among patients with cardiac arrest on admission in all LOC (all *p* < 0.001).

**Figure 8:**
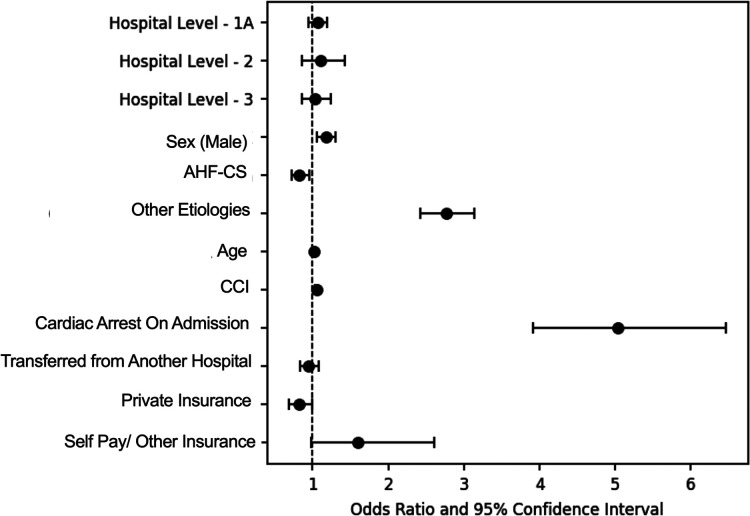
Forest plot of factors associated with hospital mortality for cardiogenic shock patients. After multivariable adjustment, level of care was not associated with hospital mortality. Level 1 was used as reference for LOC, male as a reference for sex category, AMI-CS as reference for etiology, and Medicare/Medicaid as reference for insurance category.

Overall survival to hospital discharge in this cohort was 61%. Unadjusted mortality increased in a stepwise fashion from higher to lower LOC and was higher among patients with CA on admission across all levels (Figure 7). After adjustment for age, sex, etiology of CS, Charlson Comorbidity Index, cardiac arrest on admission, admission type, and insurance type, the odds for in-hospital mortality across LOC, with Level 1 as referent, were not significantly different; Level 1A: aOR: 1.06 (95% CI: 0.95–1.19; *p* = 0.3), Level 2: aOR: 1.10 (95% CI 0.86–1.42; *p* = 0.4), and Level 3: aOR: 1.03 (95% CI:0.86–1.24; *p* = 0.7) (Figure 8).

The factors most strongly associated mortality were: cardiac arrest on admission (aOR: 5.02, 95% CI: 3.9–6.46, *p* < 0.001), having a non-AMI/non-AHF or “other” etiology of CS (aOR: 2.76; 95% CI: 2.20–3.14; *p* < 0.001) and having non-traditional insurance or being self-pay (aOR: 1.77; 95% CI: 1.07–2.77; *p* = 0.03) (Table 3).

**Table 3:** Multivariable analysis of predictors of in-hospital mortality.

	Adjusted odds ratio	95% confidence interval	*p*-value
Hospital level—1A	1.06	0.9499–1.1953	0.2784
Hospital level—2	1.106	0.8587–1.4239	0.4357
Hospital level—3	1.03	0.8619–1.2406	0.7188
Sex	1.17	1.0586–1.3030	0.0024*
AHF-CS	0.83	0.7174–0.9526	0.0085*
Other etiologies	2.76	2.4271–3.1433	<0.0001*
Age	1.02	1.0165–1.0250	<0.0001*
CCI	1.05	1.0367–1.0679	<0.0001*
Cardiac arrest on admission	5.03	3.9122–6.4641	<0.0001*
Transferred from another hospital	0.95	0.8295–1.0794	0.4110
Private insurance	0.83	0.6879–0.9945	0.0436*
Self-pay/other insurance	1.60	0.9781–2.6098	0.0613

* Signals statistically significant *p*-values.

**Figure 9:**
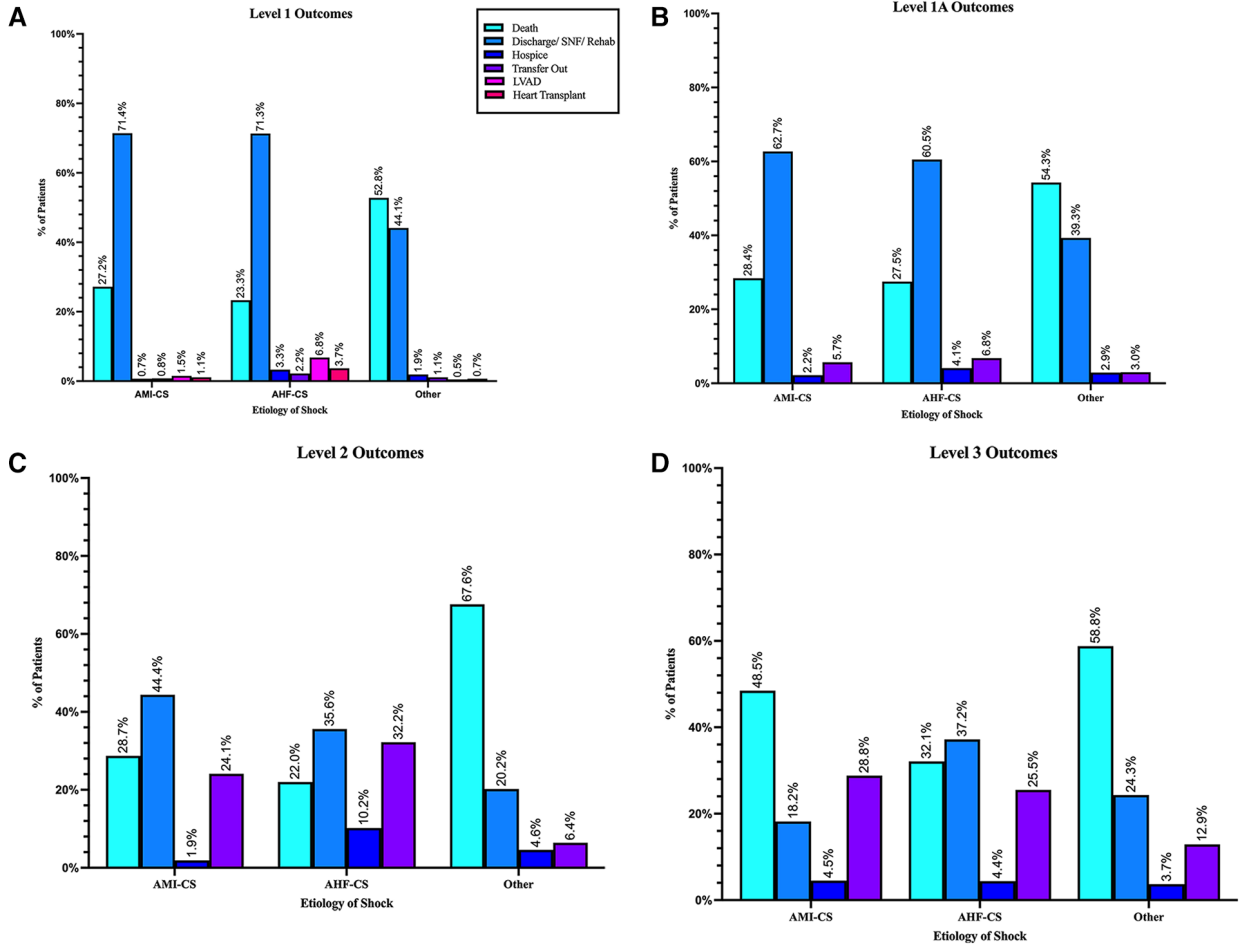
Hospital outcomes by cardiogenic shock etiology: (**A**) level 1 LVAD-capable center, (**B**) level 1A ECMO-capable centers, (**C**) level 2 pVAD-capable centers, and (**D**) level 3 no MCS capability.

Unadjusted rates of outcomes at discharge in L-1 were 35% death, 2% discharged to hospice. 3% received LVAD implantation, and 1.9% received heart transplantation; 1.4% were transferred to another hospital. Discharges to home health, skilled nursing, or other rehabilitation facilities were 48%, while routine discharges to home were only 13% (see Table 4 and Figure 9A).

**Table 4:** In-hospital outcomes.

	Level 1 (*n* = 2,830)	Level 1A (*n* = 3,452)	Level 2 (*n* = 340)	Level 3 (*n* = 780)	*p*-value
Overall mortality	34.6%	38.7%	47.4%	53.2%	<0.0001
AMI					
Death, *n* (%)	241 (27.2%)	273 (28.4%)	31 (28.7%)	32 (48.5%)	0.0033
Discharge/SNF/Rehab	633 (71.4%)	603 (62.7%)	48 (44.4%)	12 (18.2%)	<0.0001
Hospice	6 (0.7%)	21 (2.2%)	2 (1.9%)	3 (4.5%)	0.0135
Transfer out	7 (0.8%)	55 (5.7%)	26 (24.1%)	19 (28.8%)	<0.0001
LVAD	13 (1.5%)	NA	NA	NA	
Transplant	10 (1.1%)	NA	NA	NA	
AHF					
Death, *n* (%)	226 (23.3%)	299 (27.5%)	13 (22.0%)	44 (32.1%)	0.0442
Discharge/SNF/Rehab	692 (71.3%)	659 (60.5%)	21 (35.6%)	51 (37.2%)	<0.0001
Hospice	32 (3.3%)	45 (4.1%)	6 (10.2%)	6 (4.4%)	0.0634 (NS)
Transfer	21 (2.2%)	74 (6.8%)	19 (32.2%)	35 (25.5%)	<0.0001
LVAD	66 (6.8%)	NA	NA	NA	
Transplant	36 (3.7%)	NA	NA	NA	
Other etiologies					
Death, *n* (%)	513 (52.8%)	761 (54.3%)	117 (67.6%)	339 (58.8%)	0.0009
Discharge/SNF/Rehab	429 (44.1%)	551 (39.3%)	35 (20.2%)	140 (24.3%)	<0.0001
Hospice	18 (1.9%)	41 (2.9%)	8 (4.6%)	21 (3.7%)	0.0766 (NS)
Transfer	11 (1.1%)	42 (3.0%)	11 (6.4%)	75 (12.9%)	<0.0001
LVAD	5 (0.5%)	NA	NA	NA	
Transplant	7 (0.7%)	NA	NA	NA	

SNF, short-term nursing facility.

In L-1A centers mortality was 39%, 3.1% discharged to hospice, and 3% were referred to other hospitals. Discharges to home health, skilled nursing, or other rehabilitation facilities were 38%, while discharges to home were 16.8% (see Table 4 and Figure 9B).

In L-2 centers, in-hospital mortality was 47.4%, highest among patients with CS etiologies not related to AHF or AMI. Another 4.7% went to hospice. Discharges to home health, skilled nursing, or other rehabilitation facilities were 19%, while 11.5% were discharged routinely to home. Overall, 16.5% were referred to other hospitals (see Table 4 and Figure 9C).

In L-3 centers, in-hospital mortality was 53%, while 3.8% were discharged to hospice. Transfers to other hospitals were 16.5% with the highest rate of transfer among AMI-CS patients.

Discharges to home health, skilled nursing, or other rehabilitation facilities were 21%, and routine discharges to home were only 5% (see Table 4 and Figure 9D).

## 4. Discussion

**Figure 10:**
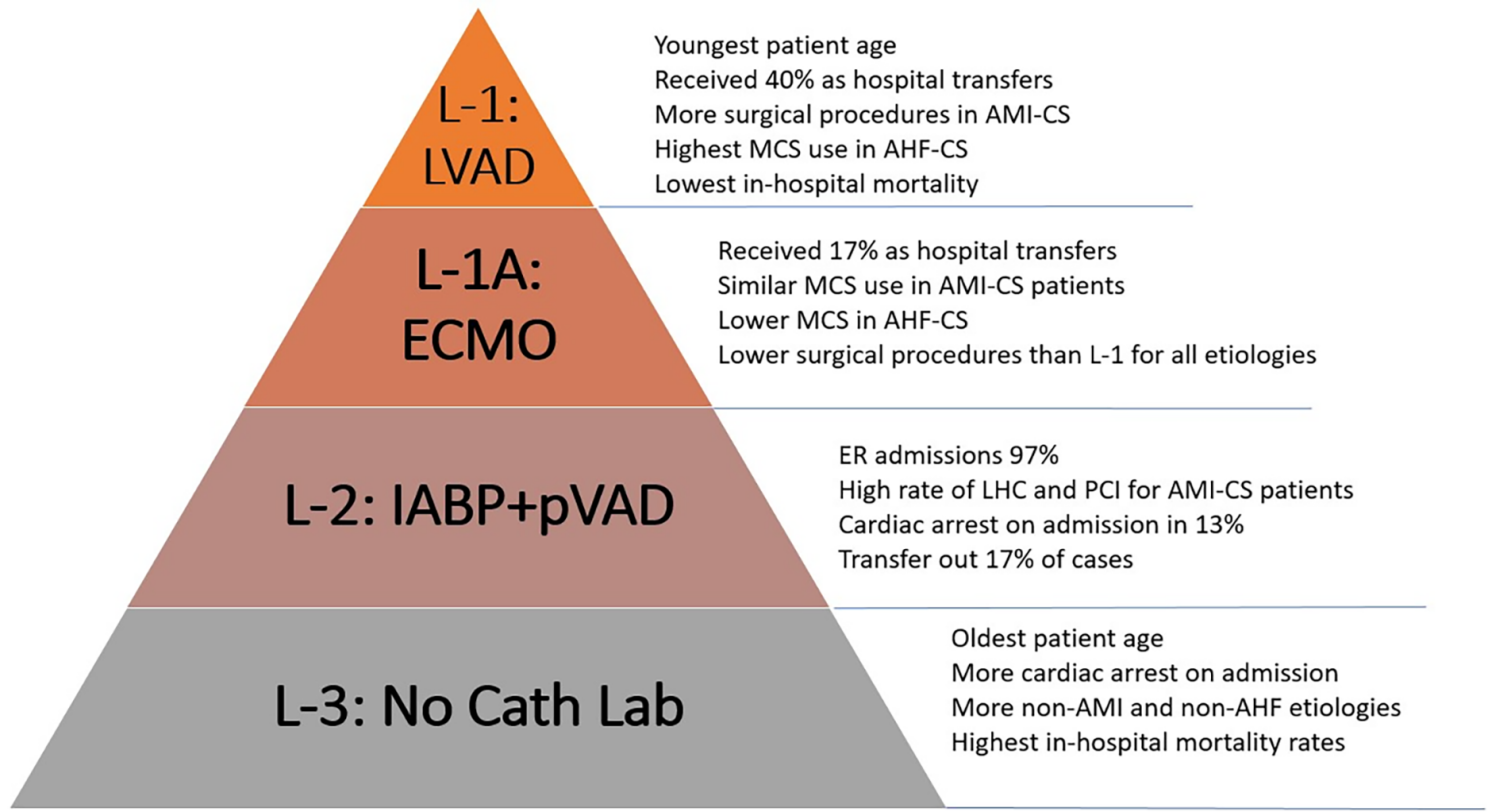
Levels of care for cardiogenic shock defined by highest MCS capability. Important differences in demographics, management strategies and outcomes were seen across different levels. Main characteristics of each level are summarized here.

To our knowledge, this is the first report to describe a pragmatic definition of CS LOC and describe the epidemiology, management, and outcomes for each different level. This classification, based on highest MCS capabilities, effectively discriminated among LOC with significant differences in patient characteristics, management strategies, and in-hospital outcomes (Figure 10). Importantly, our analysis was not restricted by admission units and included patients treated without and with all MCS devices. The observed prevalence of different CS etiologies and the overall mortality is in line with other contemporary registries (1).

Our observations suggest that surgical centers with ECMO capability serve as natural hubs for CS receiving an important proportion of patients as transfers from other hospitals. This effect is most apparent in the L-1, LVAD- and OHT-capable centers, where close to 40% of admissions came as transfers from other hospitals and patients received the highest rates of surgical and transcatheter valve procedures with lower rates of PCI. These patients were likely transferred to this LOC to receive more advanced procedures. Patients in this level had the lowest nominal mortality rates across time. Although only a small minority of them received LVAD or OHT, the beneficial effect of an in-house advanced therapy program could play a vital role in improving outcomes (13). The absence of higher survival after multivariable adjustment suggests that patient selection, either through pre-hospital triage or hospital transfers, plays an important role in improving survival at this LOC.

Although these trends were less marked for L-1A centers, hospitals in this LOC also serve as important hubs within the system despite a large range of bed capacities. They received 17% of patients from other hospitals and performed high rates of CABG and valvular procedures with comparable in-hospital survival rates. This indicates this type of center can help offload the L-1 within a multi-tier structure while serving as important receiving centers for CS patients from lower LOC.

Lower LOC, on the other hand, receive practically all their CS patients as ER admissions and have the highest rates of CA on admission, the strongest independent predictor of mortality (L-2 = 13% and L-3 = 12%). Notably, the L-2, MCS capable without ECMO, performed higher rates of LHC and PCI for patients with AMI-CS than L-1A and L-1. This is in line with prior reports that have suggested that in urban environments, small non-teaching hospitals provide similar rates of early angiography and revascularization as larger hospitals (3). Patients with non-ischemic etiologies of CS, on the other hand, received much less aggressive treatment at lower care levels, including less PAC and MCS.

Notably, close to half of patients with CS at L-2 were classified as having “other” etiologies of CS, that is, not related to AMI or AHF. This etiologic group named “other”—of which sepsis was the most prevalent singular diagnosis—was even more common among patients treated in L-3 centers, with no CCL or MCS capability. It represents a poorly understood group of patients who had the highest mortality across all LOC and received the lowest rates of management procedures, including PAC. Patients in this etiologic group tended to be more commonly women and had older average age. The characteristics of this sub-group labeled as sepsis need to be further investigated with more granular patient-level data to discern whether it represents the increasingly recognized mixed shock population reported in other cohorts of CS (1).

In L-3, otherwise, the prevalence of AMI-CS decreased over time while non-ischemic etiologies became more common. Patients in this level were also consistently older across all etiologies, had the lowest LOS, a high rate of CA, and the highest unadjusted in-hospital mortality rates. When adjusting for important risk modifiers for mortality in CS such as age, CA on admission, shock etiology, and CCI, these differences in mortality dissipated. These characteristics could largely represent an effect of pre-hospital patient triaging by emergency medical services and the effect of inter-hospital transfer of younger patients toward higher LOC.

The largest difference in non-surgical procedures across LOC was seen in the usage of PAC. L-1 and L-1A centers used PAC at rates comparable to those reported in cohorts of large academic centers (14), while the lower LOC used PAC at much lower rates for all etiologies. In each LOC, PAC usage remained mostly stable over the study period (Figure 5B).

The usage of MCS, particularly for AHF-CS, decreased with decreasing levels of care.

The effect of transfers of patients with this etiology toward the L-1 center likely explains some of these differences since close to a third of patients transferred to L-1 received MCS after arrival. An analysis of MCS escalation before and after transfer is needed to clarify this point.

## 5. Limitations

Our study relies on the accuracy of administrative data to identify patients with cardiogenic shock. However, multiple contemporary CS registries have used a similar approach, and prior studies examining the accuracy of the R57.0 ICD-10 code to identify CS have reported its positive predictive value to be as high as 93% (15).

We also rely on administrative data to classify the different CS etiologies and collect their relevant management procedures. The accuracy of these codes is less well-studied.

An additional limitation is that the effect of cardiogenic shock teams within the LOC construct could not be assessed in this report, given that they were not present in our system until late in the study period, in 2020.

Finally, the absence of patient-level data does not allow us to accurately identify patients affected with post-cardiotomy shock or to stratify patients by shock severity using the Society of Cardiovascular Angiography & Interventions (SCAI) stages of CS classification. This limits our ability to provide an accurate risk-adjustment for patients at each LOC.

## 6. Conclusion

A pragmatic definition of CS LOC based on each center's capability to deliver different types of MCS accurately discriminated center types with different epidemiology, practice patterns, and in-hospital outcomes. The differences in management across the different CS etiologies go beyond the frequency in MCS use. Care seems to be less homogeneous across LOC for non-ischemic etiologies of CS. This pragmatic definition of LOC can be applied for future research or the future creation of regional systems of care for the treatment of CS.

## Data Availability

The raw data supporting the conclusions of this article will be made available by the authors, without undue reservation.

## References

[1] Berg DD Bohula EA Van Diepen S Katz JN Alviar CL Baird-Zars VM Epidemiology of shock in contemporary cardiac intensive care units: data from the critical care cardiology trials network registry. Circ Cardiovasc Qual Outcomes. (2019) 12:e005618. 10.1161/CIRCOUTCOMES.119.005618 30879324 PMC11032172

[2] Berg DD Bohula EA Morrow DA. Epidemiology and causes of cardiogenic shock. Curr Opin Crit Care. (2021) 27:401–8. 10.1097/MCC.0000000000000845 34010224

[3] Vallabhajosyula S Dunlay SM Barsness GW Rihal CS Holmes DR Jr Prasad A. Hospital-level disparities in the outcomes of acute myocardial infarction with cardiogenic shock. Am J Cardiol. (2019) 124:491–8. 10.1016/j.amjcard.2019.05.038 31221462

[4] Van Diepen S Katz JN Albert NM Henry TD Jacobs AK Kapur NK Contemporary management of cardiogenic shock: a scientific statement from the American Heart Association. Circulation. (2017) 136:e232–68. 10.1161/CIR.0000000000000525 28923988

[5] Dhruva SS Ross JS Mortazavi BJ Hurley NC Krumholz HM Curtis JP Use of mechanical circulatory support devices among patients with acute myocardial infarction complicated by cardiogenic shock. JAMA Netw Open. (2021) 4:e2037748. 10.1001/jamanetworkopen.2020.37748 33616664 PMC7900859

[6] Sanaiha Y Bailey K Downey P Seo Y-J Aguayo E Dobaria V Trends in mortality and resource utilization for extracorporeal membrane oxygenation in the United States: 2008–2014. Surgery. (2019) 165:381–8. 10.1016/j.surg.2018.08.012 30253872

[7] Rab T Ratanapo S Kern KB Basir MB McDaniel M Meraj P Cardiac shock care centers: JACC review topic of the week. J Am Coll Cardiol. (2018) 72:1972–80. 10.1016/j.jacc.2018.07.074 30309475

[8] Tchantchaleishvili V Hallinan W Massey HT. Call for organized statewide networks for management of acute myocardial infarction–related cardiogenic shock. JAMA Surg. (2015) 150:1025–6. 10.1001/jamasurg.2015.2412 26375168

[9] Tehrani BN Sherwood MW Rosner C Truesdell AG Ben Lee S Damluji AA A standardized and regionalized network of care for cardiogenic shock. Heart Fail. (2022) 10:768–81. 10.1016/j.jchf.2022.04.004 PMC10404382 36175063

[10] Kapur NK Kanwar M Sinha SS Thayer KL Garan AR Hernandez-Montfort J Criteria for defining stages of cardiogenic shock severity. J Am Coll Cardiol. (2022) 80:185–98. 10.1016/j.jacc.2022.04.049 35835491

[11] Sundararajan V Henderson T Perry C Muggivan A Quan H Ghali WA. New ICD-10 version of the Charlson comorbidity index predicted in-hospital mortality. J Clin Epidemiol. (2004) 57:1288–94. 10.1016/j.jclinepi.2004.03.012 15617955

[12] Jentzer JC Diepen SV Barsness GW Henry TD Menon V Rihal CS Cardiogenic shock classification to predict mortality in the cardiac intensive care unit. J Am Coll Cardiol. (2019) 74:2117–28. 10.1016/j.jacc.2019.07.077 31548097

[13] Wang JI Lu DY Feldman DN McCullough SA Goyal P Karas MG Outcomes of hospitalizations for cardiogenic shock at left ventricular assist device versus non–left ventricular assist device centers. J Am Heart Assoc. (2020) 9:e017326. 10.1161/JAHA.120.017326 33222608 PMC7763759

[14] Kadosh BS Roswell R Delicce A Berg D Van Diepen S Bohula EA Use of pulmonary artery catheters in cardiac intensive care units: analysis from the critical care cardiology trials network (CCCTN) registry. Circulation. (2019) 140:A13196. 10.1016/j.jchf.2023.04.007

[15] Lauridsen MD Gammelager H Schmidt M Nielsen H Christiansen CF. Positive predictive value of International Classification of Diseases, 10th revision, diagnosis codes for cardiogenic, hypovolemic, and septic shock in the Danish national patient registry. BMC Med Res Methodol. (2015) 15:1–7. 10.1186/s12874-015-0013-2 25888061 PMC4373092

