# Supplementary material for: Defining levels of care in cardiogenic shock
Source: Front Cardiovasc Med. 2023 Oct 31;10:1206570. doi: 10.3389/fcvm.2023.1206570 (PMC10644172; doi:10.3389/fcvm.2023.1206570)
Supplement: Supplementary file 1 [file Datasheet1.pdf]

Supplementary Material

Table S1. ICD-10 Admission & Principal Diagnosis Codes Included in Each Etiology Group. *ICD, international classification of disease; AMI, acute myocardial infarction; AHF, acute heart failure; ARDS, acute respiratory distress syndrome.*

<table>
<tr><th>Etiology</th><th colspan="2">ICD-10 Diagnosis Codes</th></tr>
<tr><td>AMI</td><td colspan="2">I20.0, I20.9, I21.01, I21.02, I21.09, I21.11, I21.19, I21.21, I21.29, I21.3, I21.4, I21.9, I21.A1, I21.A9, I22.2, I24.9, I25.10, I25.110, I25.118, I25.119, I25.5, I25.9, I20.8, I22.0, I22.8, I24.8, I25.810, I23.0, I23.2, I23.3, I23.8, I24.0, I25.42, R93.1, T82.855A, T82.857A, T82.867A, Z82.49, Z98.61, R07.89, R07.9</td></tr>
<tr><td rowspan="3">AHF</td><td>Heart Failure</td><td>I50.9, I09.81, I11.0, I13.0, I13.2, I50.1, I50.20, I50.21, I50.22, I50.23, I50.30, I50.31, I50.33, I50.40, I50.41, I50.43, I50.810, I50.811, I50.813, I50.814, I50.82, I97.130, I97.131, J81.0, J81.1, I50.84, I31.9, I40.0, I40.9, I42.0, I42.2, I42.8, I42.9, I51.4, I51.7, I51.81, O90.3, O99.42, Z94.1, Z95.811, B57.2, I40.8, I42.6, I42.7, O99.43</td></tr>
<tr><td>Arrythmia</td><td>I47.1, I47.2, I48.0, I48.1, I48.19, I48.20, I48.3, I48.91, I48.92, I49.01, I49.3, I49.5, I49.8, I49.9, R00.0, R00.1, R00.2, I48.21, I44.1, I45.5, I45.9, R94.31, I44.2, I44.30</td></tr>
<tr><td>Valvular Disorders</td><td>I05.0, I05.1, I05.9, I06.0, I07.1, I08.0, I08.1, I08.3, I34.0, I34.1, I34.8, I34.9, I35.0, I35.1, I35.2, I35.9, Q23.0, Q23.1, I05.2, I33.0, I33.9, I34.2, I35.8, I36.1, I37.1, I38 T82.03XA, T82.09XA, T82.228A, T82.6XXA, T82.01XA, T82.221A, T82.310A</td></tr>
<tr><td rowspan="8">Other</td><td>Sepsis/ Infection</td><td>A09, A32.7, A40.0, A40.1, A40.3, A40.8, A40.9, A41.01, A41.02, A41.1, A41.3, A41.4, A41.50, A41.51, A41.52, A41.53, A41.59, A41.81, A41.89, A41.9, A48.1, A49.9, B02.7, B19.20, B34.9, J06.9, J12.1, J12.9, J15.8, J15.9, J18.1, J18.9, J20.9, J85.1, O98.52, R65.21, T80.211A, T80.212A, T81.42XA, T81.44XA, U07.1, B20, B33.8, B37.7, J10.01, J10.08, J10.1, J12.89, J15.211, J15.6, J20.8, T80.218A, T80.29XA, T81.41XA, T81.43XA, T81.4XXA, T83.511A, T83.512A, T83.518A, T83.592A</td></tr>
<tr><td>Surgical</td><td>I71.00, I71.01, I71.02, I71.03, I71.1, I71.2, I71.3, I71.4, I71.6, I71.8, I71.9, I74.09, I74.11, I77.810, I97.190, I97.51, I97.621, I97.790, I97.88, I97.89, Q25.43, Q25.49, T82.330A</td></tr>
<tr><td>Pulmonary Embolism</td><td>I23.6, I26.02, I26.09, I26.90, I26.92, I26.93, I26.94, I26.99, I27.82, I82.220, I82.403, I82.409, I82.432, I82.441, I82.4Z2, I82.622, I87.8</td></tr>
<tr><td>Cardiac Arrest</td><td>I46.8, I46.9, I97.120, I97.121, I97.711</td></tr>
<tr><td>ARDS</td><td>J80, J96.00, J96.01, J96.02, J96.20, J96.21, J96.22, J96.90, J96.91, J96.92, J98.9, R06.03, R09.2</td></tr>
<tr><td>Kidney Disease</td><td>I12.0, I12.9, I13.10, N17.0, N17.8, N17.9, N18.3, N18.6, N18.9, N19</td></tr>
<tr><td>Other Cardiac Diagnosis</td><td>D15.1, I25.3, I27.9, I30.8, I30.9, I31.1, I31.2, I31.3, I31.4, I51.1, I51.3, I51.89, I51.9, I73.9, I74.3, I74.2, I74.5, I74.9, I77.9, I99.8, M32.12, O9A.12, Q21.0, Q21.1, Q21.3, Q23.4, R09.89, R94.30, R94.39, T82.110A, T82.118A, T82.120A, T82.190A, T82.198A, T82.41XA, T82.42XA, T82.518A, T82.524A, T82.594A, T82.598A, T82.818A, T82.838A, T82.897A, T82.898A, T82.9XXA, Z45.010, Z45.02, Z95.810</td></tr>
<tr><td>Other Non-Cardiac Diagnosis</td><td>000.00, A04.72, A15.0, A24.9, A26.7, A48.0, B01.11, B19.9, B37.81, B96.5, C13.9, C15.4, C15.9, C16.8, C17.0, C18.0, C18.4, C18.6, C18.7, C18.9, C19, C21.8, C25.0, C25.2, C34.02, C34.11, C34.12, C34.90, C34.91, C34.92, C38.0, C40.21, C48.0, C49.3, C54.1, C55, C61, C64.1, C67.9, C70.1, C71.9, C77.1, C78.00, C78.01, C78.6, C78.7, C79.31, C79.51, C7A.1, C80.1, C82.90, C83.30, C83.32, C83.39, C84.48, C85.10, C85.11, C85.18, C85.89, C85.90, C85.91,C85.95, C86.5, C90.00, C91.00, C91.10,C91.50, C91.51, C91.92, C92.00, C92.Z0, C95.90, D32.0, D35.01, D35.1, D35.2, D3A.090, D3A.8, D46.9, D50.0, D57.00, D57.01, D59.1, D59.13, D61.810, D61.818, D62, D64.9, D65, D68.32, D68.9, D69.3, D69.6, D70.9, D72.829, D86.0, D86.89, E03.5, E05.01, E10.10, E11.00, E11.10, E11.22, E11.43, E11.51, E11.52, E11.621, E11.649, E11.65, E11.69, E15, E16.2, E22.2, E32.8, E66.01, E72.20, E80.6, E83.42, E83.51, E83.52, E83.59, E85.2, E85.4, E85.81, E85.82, E86.0, E86.9, E87.0, E87.1, E87.2, E87.5, E87.70, E87.79, E87.8, E88.09, E88.3, F10.10, F10.120, F10.221, F10.231, F10.239, G00.8, G04.81, G06.2, G40.101, G40.401, G40.89, G40.901, G40.909, G45.1, G45.9, G58.8, G70.01, G89.11, G92, G93.1, G93.40, G93.41, G93.49, G93.6, G93.89, G95.29, H50.07, H53.9, I10, I16.0, I16.1, I27.0, I27.20, I27.21, I27.24, I27.29, I28.8, I60.00, I60.11, I60.2, I60.31, I60.4, I60.7, I60.8, I60.9, I61.1, I61.5, I61.8, I61.9, I62.00, I62.01, I62.9, I63.12, I63.211, I63.212, I63.231, I63.233, I63.311, I63.40, I63.411, I63.433, I63.50, I63.511, I63.512, I63.519, I63.521, I63.59, I63.89, I63.9, I65.1, I65.22, I65.29, I67.1, I67.89, I69.398, I70.201, I70.203, I70.211, I70.222, I70.232, I70.234, I70.261, I70.421, I72.4, I72.9, I77.71, I77.74, I81, I85.01, I96, J36, J39.8, J43.9, J44.0, J44.1, J45.51, J45.901, J45.902, J68.1, J69.0, J69.8, J70.2, J84.10, J84.112, J85.2, J86.0, J86.9, J90, J91.8, J93.0, J93.9, J94.2, J95.03, J95.811, J95.830, J95.89, J98.4, J98.51, J98.59, J98.8, K11.20, K11.21, K20.91, K22.3, K25.1, K25.4, K25.5, K25.6, K26.0, K26.4, K26.5, K26.6, K29.01, K31.1, K31.811, K31.82, K35.80, K40.30, K41.40, K42.0, K43.0, K43.1, K43.2, K43.3, K43.5, K43.6, K43.9, K45.0, K45.8, K46.0, K52.9, K55.019, K55.039, K55.059, K55.1, K55.21, K55.8, K55.9, K56.1, K56.2, K56.41, K56.5, K56.50, K56.60, K56.601, K56.609, K56.69, K56.691, K56.7, K57.20, K57.31, K57.33, K57.40, K57.91, K57.92, K58.0, K59.00, K62.5, K62.89, K63.1, K65.1, K65.9, K66.1, K66.8, K70.30, K70.31, K70.40, K70.9, K71.10, K71.11, K72.00, K72.01, K72.90, K74.60, K74.69, K75.0, K76.1, K76.7, K80.00, K80.20, K80.30, K80.31, K80.50, K80.71, K81.0, K81.9, K82.8, K83.09, K83.3, K85.10, K85.12, K85.20, K85.21, K85.90, K86.1, K91.840, K92.0, K92.1, K92.2, K92.9, K95.89, L02.214, L02.511, L02.91, L03.115, L03.116, L03.119, L03.90, L08.9, L29.9, L76.22, L76.34, L89.150, L89.159, L97.101, L97.509, L97.519, L98.492, M00.011, M10.9, M17.11, M17.12, M25.511, M25.519, M25.551, M25.561, M31.1, M33.12, M35.81, M35.9, M40.205, M40.209, M43.9, M46.27, M48.06, M48.061, M48.062, M48.56XA, M50.20, M51.16, M54.16, M54.2, M54.9, M62.82, M66.862, M71.551, M72.6, M79.602, M79.604, M79.605, M79.606, M79.609, M79.662, M79.671, M79.89, M80.052A, M80.88XA, M84.40XA, M84.551A, M84.552A, M84.562A, M84.58XA, M86.10, M86.171, M86.642, M89.18, M97.11XA, N05.8, N10, N12, N13.6, N14.1, N20.0, N25.9, N28.9, N30.01, N30.90, N32.1, N32.89, N36.5, N39.0, N39.3, N48.89, N49.3, N76.89, N81.3, N82.3, N82.4, N92.0, N93.9, N95.0, O75.3, O9A.212, P76.9, Q27.33, Q43.0, R04.0, R04.2, R04.89, R05, R06.2, R06.89, R09.02, R10.11, R10.13, R10.31, R10.84, R10.9, R11.0, R11.10, R11.15, R11.2, R13.10, R13.13, R14.0, R17, R18.8, R19.00, R19.01, R19.7, R22.2, R22.43, R23.0, R25.1, R26.2, R26.81, R26.89, R26.9, R29.6, R29.810, R29.898, R29.90, R31.0, R31.9, R40.1, R40.20, R40.4, R41.0, R41.82, R41.89, R42, R47.01, R47.1, R47.81, R50.9, R51, R52, R53.1, R53.81, R53.83, R55, R56.1, R56.9, R57.1, R57.8, R57.9, R58, R59.1, R60.0, R60.1, R60.9, R62.7, R63.0, R63.8, R68.0, R68.89, R73.9, R74.0, R74.01, R74.8, R77.8, R78.81, R79.1, R79.89, R91.1, R91.8, R94.8, S00.83XA, S01.81XA, S02.2XXA, S02.80XA, S06.339A, S06.4X0A, S06.5X0A, S06.5X9A, S06.6X0A, S06.6X9A, S09.90XA, S12.111A, S12.120A, S15.121A, S21.431A, S22.009A, S22.069A, S22.081A, S22.20XA, S22.31XA, S22.39XA, S22.41XA, S22.42XA, S22.43XA, S22.49XA, S27.1XXA, S32.19XA, S32.511A, S32.591A, S32.599A, S32.810A, S36.031A, S36.032A, S36.899A, S39.91XA, S42.201K, S46.221A, S70.11XA, S72.001A, S72.002A, S72.009A, S72.012A, S72.021A, S72.041A, S72.091A, S72.092A, S72.111A, S72.141A, S72.142A, S72.145A, S72.21XA, S72.341A, S72.401A, S72.452A, S72.491A, S72.492A, S72.91XA, S73.005A, S76.112A, S80.11XA, S82.109C, S82.142C, S82.252A, S82.842C, S82.892B, T07, T14.8, T14.8XXA, T14.90, T14.90XA, T14.91XA, T17.820A, T17.890A, T17.908A, T17.920A, T17.928A, T22.391A, T24.311A, T30.0, T40.1X1A, T40.1X4A, T40.2X1A, T40.3X2A, T40.5X1A, T42.1X1A, T42.4X1A, T42.4X2A, T42.4X4A, T43.212A, T43.591A, T43.594A, T43.621A, T44.7X1A, T44.7X2A, T44.8X1A, T44.901A, T45.0X2A, T45.511A, T46.0X1A, T46.1X1A, T46.1X1S, T46.1X2A, T46.5X1A, T46.5X2A, T50.4X2A, T50.4X4A, T50.901A, T50.902A, T50.991A, T53.6X4A, T56.891A, T67.5XXA, T68.XXXA, T71.162A, T71.164A, T78.2XXA, T78.3XXA, T79.4XXA, T79.6XXA, T79.A0XA, T79.A21A, T79.A22D, T81.30XA, T81.31XA, T81.32XA, T81.595A, T81.718A, T81.719A, T81.83XA, T81.89XA, T81.9XXA, T84.125A, T85.611A, T85.868A, T86.19, T86.5, T87.43, T87.44, T87.54, T87.89, T88.6XXA, V87.7XXA, Z04.3, Z20.828, Z34.80, Z41.8, Z45.2, Z47.33, Z51.11, Z51.5, Z51.89, Z68.42, Z82.3, Z86.711, Z90.10, Z92.81</td></tr>
</table>

Table S2. ICD-10 Procedure Codes. *LHC, left heart catheterization; RHC, right heart catheterization; SWAN, swan-ganz catheterization; PCI, percutaneous coronary intervention; CABG, coronary artery bypass graft; VA ECMO, Veno-arterial extracorporeal membrane oxygenation; MCS, percutaneous temporary mechanical circulatory support; IABP, intra-aortic balloon pump; pVAD, percutaneous ventricular assist device, LVAD, left ventricular assist device*

Procedure	ICD-10 Procedure Codes	
**LHC**	**4A023N7, 4A023N8, B2111ZZ, B211YZZ, B2161ZZ, B216YZZ, B2151ZZ, B215YZZ, B2181ZZ, B218YZZ, B2131ZZ, B213YZZ, B21F1ZZ, B2121ZZ, B2101ZZ, B210110**	
**RHC/SWAN**	**4A023N6, 4A023N8, B2141ZZ, B31S1ZZ, B31U1ZZ, B214YZZ, B2161ZZ, B216YZZ, B2171ZZ, 5A12012, B31TYZZ, 02HR32Z, 02HQ33Z, 02HR33Z, 02HP32Z, 02HQ02Z, 02HQ32Z, 4A133B3, 5A1221Z**	
**PCI**	**027337Z, 027037Z, 027036Z, 02703FZ, 027035Z, 02703EZ, 0270346, 0270356, 027034Z, 0270456, 02703DZ, 02703Z6, 02703ZZ, 027237Z, 027236Z, 0272356, 0272346, 027234Z, 02723ZZ, 027137Z, 027136Z, 027135Z, 02713EZ, 0271376, 0271366, 0271356, 027134Z, 02713DZ, 02714E6, 02713ZZ, 02C03ZZ, X2C2361, 02C13Z6, X2C1361, 02C13ZZ, 0271346, 0273346, 0270366, 027046Z, 02C00ZZ**	
**CABG**	**021309W, 02130Z9, 0210093, 0210099, 02100AW, 02100KW, 02100JW, 02100J3, 02100Z3, 02100A9, 02100Z9, 02100Z8, 02100AC, 02100ZC, 0212093, 0212099, 021209W, 0211093, 0211099, 02110AW, 021109W, 021009W, 02110KW, 02110JW, 02100K8, 02110Z9, 02110Z8, 02110AC, 0213099, 02100A8, 021009C, 02120Z9, 02110A8, 02110Z3**	
**Valve Procedure**	**025G3ZZ, 027F3ZZ, 02BF0ZZ, 02BG0ZX, 02BG0ZZ, 02BJ0ZZ, 02CF0ZZ, 02CG0ZZ, 02CJ0ZZ, 02QF0ZZ, 02QG0ZZ, 02QJ0ZZ, 02RF07Z, 02RF08Z, 02RF0KZ, 02RF38Z, 02RF3JZ, 02RG08Z, 02RG0JZ, 02RG0KZ, 02RH0KZ, 02RJ08Z, 02UF08Z, 02UF0JZ, 02UF0KZ, 02UG07Z, 02UG08Z, 02UG0JZ, 02UG3JZ, 02UJ0JZ, 0DBC8ZX, X2RF032, 02RG3JH, 02RH08Z, 02RJ38Z, 02UJ3JZ, 02UJ4JZ, 02VG0ZZ, 02WG0JZ**	
**MCS**	**IABP**	**5A02210, 5A02110**
	**pVAD**	**5A0221D, 02HA3RZ, 02HA4RZ, 02HA3RJ, 02HA0RZ, 5A0211D, 02HA3RS**
	**VA ECMO**	**5A15223, 5A1522G, 5A1522F, 5A02216**
	**Protek Duo**	**02HA3QZ**
	**LVAD**	**02HA0QZ**
**Heart Transplant**	**02YA0Z0**	

Table S3. Overview of Centers Included in the Northwell Shock Registry (n=11). *AHF, advanced heart failure; CTS, cardiothoracic surgery; CCU, cardiac intensive care unit; ECMO, extracorporeal membrane oxygenation; HT, heart transplantation; MCS, percutaneous temporary mechanical circulatory support; VAD, durable ventricular assist device*

Site Code	Shock Level	Bed Capacity	MCS	CTS/ ECMO	Cath Lab	VAD/ HT	AHF Service	Dedicated CCU
**A**	1	756	yes	yes	yes	yes	yes	yes
**B**	1a	632	yes	yes	yes	no	yes	yes
**C**	1a	313	yes	yes	yes	no	no	no
**D**	1a	807	yes	yes	yes	no	yes	yes
**E**	1a	491	yes	yes	yes	no	yes	yes
**F**	2	348	yes	no	yes	no	no	yes
**G**	3	216	no	no	no	no	no	no
**H**	3	204	no	no	no	no	no	no
**I**	3	312	no	no	no	no	no	no
**J**	3	284	no	no	no	no	no	no
**K**	3	103	no	no	no	no	no	no
