# Supplementary figures and images for: Defining levels of care in cardiogenic shock
Source: Front Cardiovasc Med. 2023 Oct 31;10:1206570. doi: 10.3389/fcvm.2023.1206570 (PMC10644172; doi:10.3389/fcvm.2023.1206570)

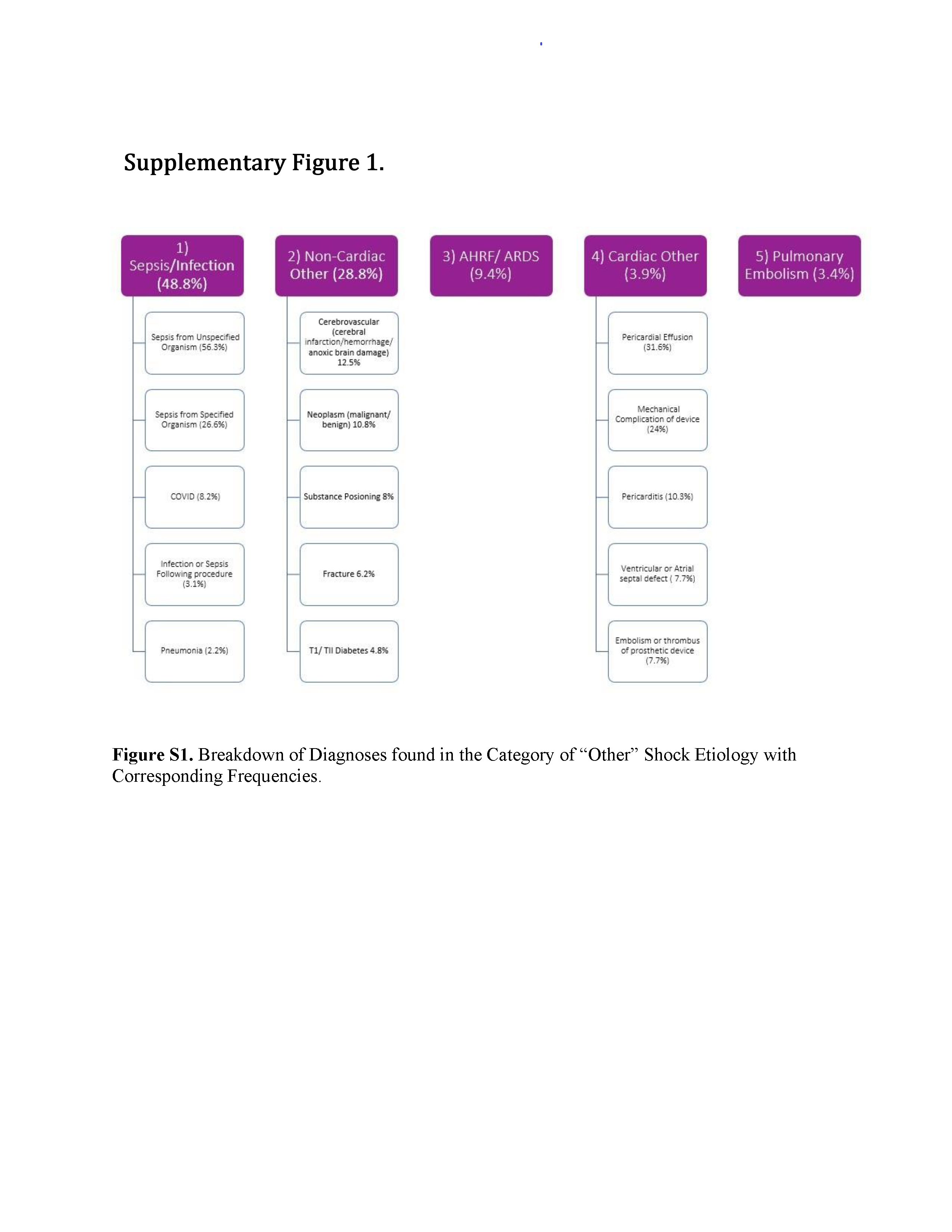

Supplement: Supplementary file 2 [file Image1.tiff]

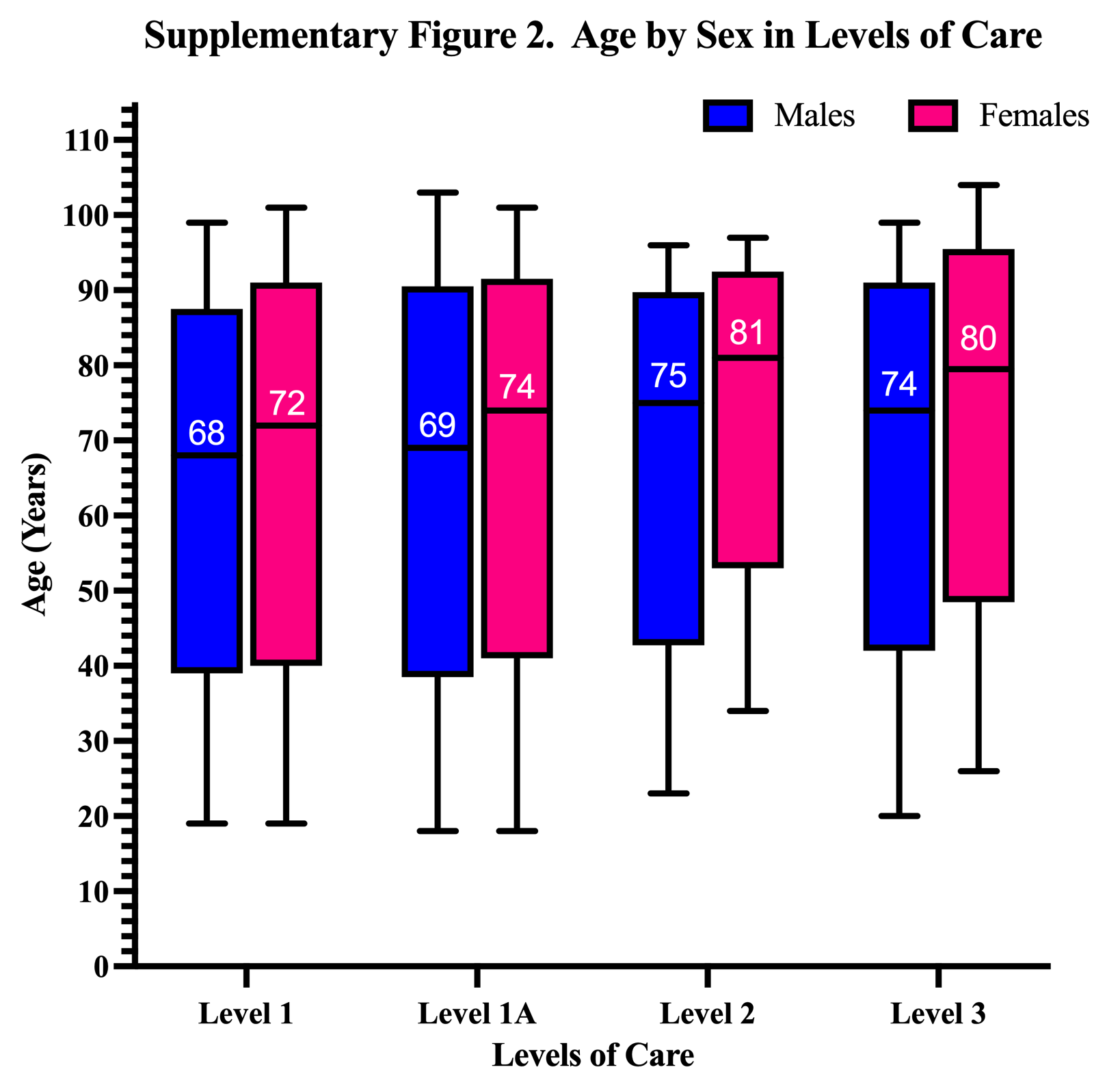

Supplement: Supplementary file 3 [file Image2.tiff]

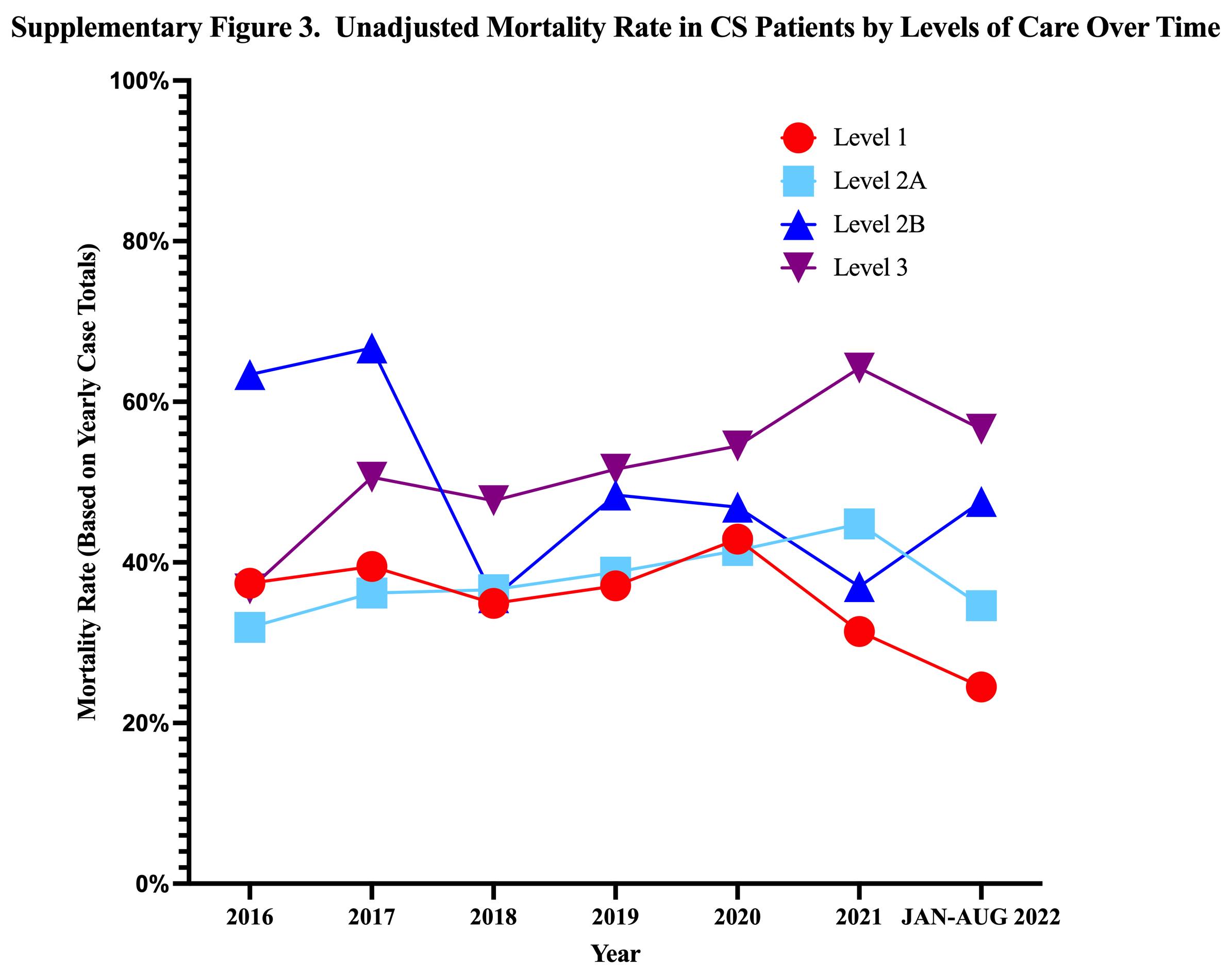

Supplement: Supplementary file 4 [file Image3.tiff]
